# Adaptive self‐organization in the embryo: its importance to adult anatomy and to tissue engineering

**DOI:** 10.1111/joa.12691

**Published:** 2017-10-10

**Authors:** Jamie A. Davies

**Affiliations:** ^1^ Deanery of Biomedical Sciences University of Edinburgh Medical School Edinburgh UK

**Keywords:** mini‐organ, organoid, symmetry, symmetry‐breaking, synthetic biology, synthetic morphology

## Abstract

The anatomy of healthy humans shows much minor variation, and twin‐studies reveal at least some of this variation cannot be explained genetically. A plausible explanation is that fine‐scale anatomy is not specified directly in a genetic programme, but emerges from self‐organizing behaviours of cells that, for example, place a new capillary where it happens to be needed to prevent local hypoxia. Self‐organizing behaviour can be identified by manipulating growing tissues (e.g. putting them under a spatial constraint) and observing an adaptive change that conserves the character of the normal tissue while altering its precise anatomy. Self‐organization can be practically useful in tissue engineering but it is limited; generally, it is good for producing realistic small‐scale anatomy but large‐scale features will be missing. This is because self‐organizing organoids miss critical symmetry‐breaking influences present in the embryo: simulating these artificially, for example, with local signal sources, makes anatomy realistic even at large scales. A growing understanding of the mechanisms of self‐organization is now allowing synthetic biologists to take their first tentative steps towards constructing artificial multicellular systems that spontaneously organize themselves into patterns, which may soon be extended into three‐dimensional shapes.

## Introduction: the paradox of anatomical variation

One of the first things that medical students learn of human anatomy, usually to their frustration, is that its details are surprisingly variable. Blood vessels can vary in route and number (Dăescu et al. 2012; Bertrand et al. 2014; Silva et al. 2014; Marco‐Clement et al. 2016; Tomaszewski et al. 2017), innervation of the same structure can differ between individuals (Matejcík, 2010; Henry et al. 2016; Wolf et al. 2016), the number of bones in the cranium can vary due to occasional appearance of wormian (intra‐sutural) bones (Hess, 1946; Ghosh et al. 2017), and there are many more examples of unpredictable anatomy in anatomical and surgical literature. Some of these differences may be specified genetically, but studies of monozygotic twins have demonstrated that histogenesis can vary even when driven by exactly the same ‘genetic programme’. This fact even finds application in the forensic sciences; while the ‘DNA fingerprints’ of monozygotic twins are the same, their literal fingerprints are readily distinguishable (Srihari et al. 2008; Kücken & Champod, 2013). Palatal rugae also differ between monozygotic twins (Herrera et al. 2017), while patterns of cortical gyrification and dentition can, but do not always, do so (Townsend et al. 2005; Kates et al. 2009). In this review, I will consider possible sources of this variation, with a particular focus on processes of self‐organization that produce anatomies that are predictable in terms of overall statistical properties but not in terms of fine detail. The review will then consider practical uses for this self‐organization, and current limitations.

Anatomical variation that cannot be explained genetically is traditionally accounted for in terms of environmental influences or developmental errors. Environmental differences can be largely discounted for anatomical features that arise during foetal development, especially between identical twins, although it is possible that the precise site of placental implantation into the uterus might affect a limited number of things. This leaves error – the notion that variation arises through inaccurate following of the genetically specified developmental programme. A simple error theory, however, runs into a problem: it is in the nature of embryonic development that the morphogenetic events of each stage build on the results of events at previous stages. Errors would therefore be cumulative and, if each event were directly programme‐driven, even a perfectly executed second mechanism could go badly wrong if it followed an earlier error. In this system, for example, a ‘perfectly’ positioned artery, in terms of textbook diagrams, would fail to bring blood to an organ if the organ's rudiment had been placed atypically through a previous error. If every stage of embryonic development was as variable as adult anatomy, and all events ran directly to a genetic programme, one would expect development of a complex body to degenerate into a chaos of collisions and missed connections. Yet it does not: that is a paradox for anyone who regards development as the direct following a set of instructions in a genetic programme.

A second paradox comes from the observation that the functional tissue elements (alveoli, islets, glomeruli, capillaries, etc.) of large and small mammals are of similar size (linear dimensions differing by no more than a factor of 3 or so); the organs of large mammals simply contain more of them, typically around 1000 times more in human than in mouse. That means thousands of times more items to be properly positioned, yet the genomes of humans and mice are of similar size, at about 3 Gigabases, containing about 19 000 protein‐coding genes (International Human Genome Sequencing Consortium, 2001; Chinwalla et al. 2002; Ezkurdia et al. 2014). The similar sizes, and the fact that there are far fewer genes than body structures, challenge the idea of genes specifying anatomy in any direct way.

A solution to the paradox, a solution that has been written about many times before (Turing, 1953; Meinhardt & Gierer, 2000; Davies, 2005; Bozorgmehr, 2014), although it has not succeeded in expunging the idea of anatomical features being under direct genetic control (‘a gene for’ a specific anatomical feature), is to view anatomy as emerging from a complex interaction of cells that use communication to organize themselves according to the conditions in which they actually find themselves. Understanding the basis of this adaptive self‐organization is therefore critical for understanding development, for understanding how variation arises, and for explaining how it can do so harmlessly. It also promises to offer valuable new approaches to tissue engineering.

## Adaptive self‐organization: constancy and change

The principles of adaptive self‐organization in development can be illustrated by the well‐known system that adapts the anatomy of capillary systems to the tissue that must be served. Tissue cells produce a transcription factor, Hif‐1α, that is unstable in the presence of oxygen (Maxwell et al. 1999; Cockman et al. 2000). Hif‐1α, when it survives, drives transcription of vascular endothelial growth factor (VEGF; Liu et al. 1995; Forsythe et al. 1996), and VEGF diffuses away and acts as an inducer of capillary sprouting and a chemoattractant for the new sprouts (Millauer et al. 1993; Yu & Sato, 1999). The effect of this is that the capillary system automatically grows and adapts to serve the needs of growing tissues that experience hypoxia by out‐growing their existing capillary supply. The amino acid sequences of the protein machines that underly this system are of course specified by genes, but the vascular anatomy that emerges from the action of those machines is not (see Davies, 2005, for more detailed discussion).

The mechanism for adaptive capillary formation described above relies on negative feedback of a type seen in many examples of physiological homeostasis (thermostat, glucostat, etc.: see Romanovsky, 2007; Röder et al. 2016 for recent reviews). The main difference is that, whereas conventional homeostatic systems have evolved to resist change, self‐organizing ones have evolved to accommodate and to drive change. When a tissue grows and acquires new capillaries, it changes quickly; what is conserved is not the physical anatomy but rather the abstract architecture of the tissue. In this case, the relevant architecture can be described in terms of density of capillaries. In the cases presented below, it can be described in terms of the relationship between different cell types at the ends of a branching tree or of the connections between different types of tubules. The concept of conserved architectures extends beyond anatomy and can be applied, for example, to proportions of stem and differentiated cell types in a population or to the energy fluxes through different metabolic pathways (Schilling et al. 1999; Rodriguez‐Brenes et al. 2013; Lavington et al. 2014; Batsivari et al. 2017).

Self‐organizing processes operate through a very wide range of organization levels, in both embryonic and adult lives. They are seen at the subcellular level in the assembly of macromolecular complexes (Misteli, 2001; Kurakin, 2005; Karsenti, 2008; Gordon et al. 2012), at the level of cell populations and tissues (Green et al. 2004; Takeichi, 2011), and extend even beyond the individual organism to populations (De Palo et al. 2007; Hölldobler & Wilson, 2008) and ecosystems (Braakmana et al. 2017). The rest of this review will, however, be restricted to the level of histogenesis.

## Identifying adaptive self‐organization

**Figure 1:**
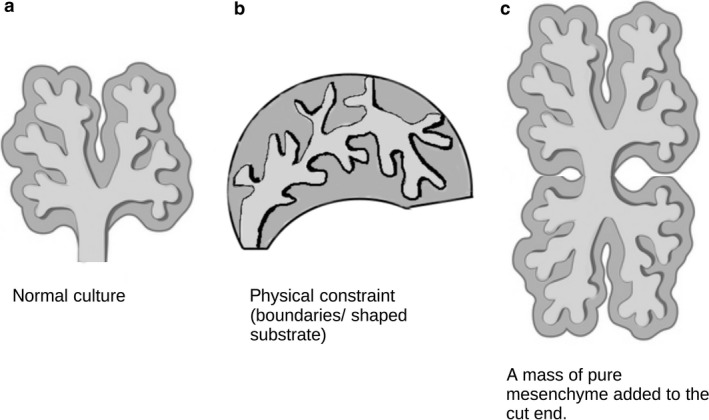
Practical approaches to seek evidence for adaptive self‐organization. Simple manipulations, such as constraining the physical shape of a tissue or adding or subtracting components, can be applied to a growing organ or tissue in culture. The ability of the system to create an anatomy that accommodates the unusual circumstances but is still recognizable as characteristic of the normal organ – for example having typical branch morphologies even if the tree is strange – is an indication of adaptive self‐organization.

There are two broad approaches for identifying instances of adaptive self‐organization, one directly experimental and the other theoretical. The practical one typically begins with observation of a developmental phenomenon that seems to show variation and adaptation either naturally or in an experimental system. These experimental systems can be very simple. To take one example, many organ rudiments (e.g. lung, salivary gland, mammary gland, prostate, kidney) can be removed from a mouse embryo and cultured on a filter at a gas‐medium interface (Trowell, 1954; Jainchill et al. 1964). Such cultures grow very flat compared with the natural organs, typically only a few tens of microns thick, yet their internal tissues form in a way usually described as ‘organotypic’: epithelial trees are still tree‐like and mesenchymal tissue surrounds them in a way that reflects its organization *in vivo*. The ability of tissues that should form very three‐dimensional organs to form flat but ‘realistic’ versions of their normal selves is a promising indication that a process of adaptive self‐organization is at work. The ability of a tissue to mitigate the effects of a perturbation such as cut‐and‐paste removal and grafting of its components is another indication (Fig. 1).

**Figure 2:**
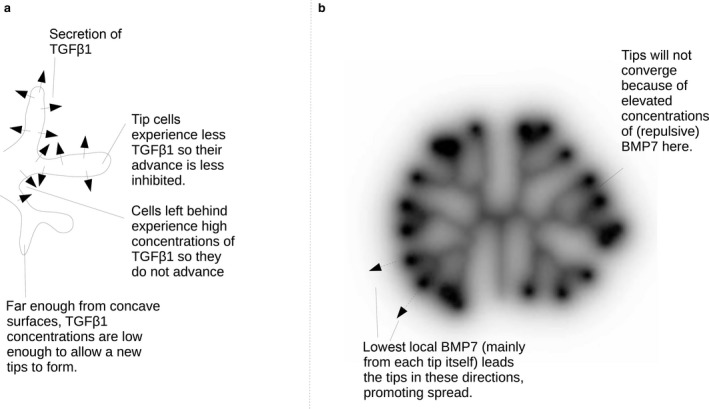
Two models for self‐organization of branching epithelial tubules at different scales, both centred on autocrine secretion of an inhibitor. (a) A sketch depicting the model of Nelson et al. (2006), for spacing out branch initiation by cells secreting a soluble inhibitor of branching, the concentration of which is depicted by grey shading. (b) A computer model of BMP7‐mediated mutual repulsion of branches, at least part‐responsible for ensuring that the collecting duct tree spreads out rather than becoming tangled (Davies et al. 2014). In (b), the depth of grey indicates calculated concentrations of BMP7.

Given that epithelial trees in organs show evidence of self‐organization, questions arise about how the system organizes itself so that some cells initiate new branches and others do not, and so that branches spread out rather than tangle. An indication that even cells that are not initiating branches *can* do so can be gained by removing the branches from a ‘stalk’, for example the presumptive ureter of a mouse ureter‐collecting duct tree, placing the stalk in appropriate mesenchyme and observing that it now initiates branching (Sweeney et al. 2008). It can also be gained from the observation that epithelial cysts in Matrigel, even clonal ones made from mouse cell lines, will produce branching trees, some cells being branch leaders and others remaining behind although all began exactly the same (Montesano et al. 1991; Tai et al. 2013). To find out why cells that can branch might nevertheless refrain from doing so, Nelson et al. (2006) cultured populations of mouse mammary epithelial cells in shaped wells in gelled matrix, and used matrix invasion as a proxy for branching. They found that cells at the extremities of long rectangular wells would be invasive but those towards the middle of the rectangle would not. This invasive behaviour could be prevented by the proximity of another well of cells. These and other, broadly similar observations suggested a model in which cells secrete a substance, probably TGFβ, which inhibits invasion. In an epithelial tree, such a system would ensure that, once a branch has started to form, cells at its tip would be running away from the inhibitor secreted by the tree so would remain highly invasive while those left behind near the branch site would experience an even higher concentration of inhibitor, as it accumulates in ‘bays’ nearly surrounded by cells secreting it. These cells will remain non‐invasive (Fig. 2a).

While the Nelson hypothesis described above is powerful, it should be noted that a recent attempt to explore it in two‐dimensional culture of canine renal collecting duct cells, in which cells were cultured on shaped islands in the presence or absence of flow, confirmed that cells on convex curves are far more invasive than cells on straight edges and those on concave curves are particularly reluctant to move. Application of flow strong enough to sweep proteins away failed to alter this relationship; however, casting doubt on the hypothesis control by accumulation of a secreted inhibitor and suggesting that curvature itself may control invasion directly, for example by its effect on cytoskeletal tension (Martin et al. 2017).

The ability of epithelial trees to accommodate themselves to unnatural environments, for example flat culture filters, and still to spread out organotypically has already been mentioned. Culture of multiple mouse ureteric bud (renal collecting duct progenitor) trees in very close proximity and on what would seem to be a collision course fails to produce collisions. Instead, each tree becomes distorted, but avoids contact with branches of the other tree. This avoidance is abrogated, however, if signalling by BMP7 is inhibited; indeed even within a tree growing in isolation, branches can collide and tangle in the absence of BMP7 signalling. Given that BMP7 and its receptor are both made by branches, these observations have suggested a model in which the tree self‐organizes by each branch growing in the direction of least BMP7, and therefore avoids growing backwards towards itself or growing towards other branches that are secreting their own BMP7 (Fig. 2b; Davies et al. 2014).

In the kidney, the tips of growing branches are surrounded by a population of mesenchymal stem cells, the ‘cap mesenchyme’ (Schreiner, 1902; Reinhoff, 1922). At least in mouse, this population depends on BMP7 and FGF9 signalling from the tip for its continued maintenance (Barak et al. 2012; Muthukrishnan et al. 2015) and, when a tip divides and its daughters become separated by new stalk, only the cap cells that surround the diverging tips retain the cap phenotype and the space between them becomes occupied by a newly sprouting blood vessel (Munro, 2017). Caps do not divide in this way when they are induced chemically in pure mesenchyme (Davies & Garrod, 1995; Kuure et al. 2007). The vessels seem to find themselves there by growing up the stalks of the developing tree (Munro, 2017). These two facts illustrate something else common in self‐organizing systems: one tissue type, in this case ureteric bud, may be a primary organizer of space, while other entities such as the cap and the blood vessels take their cues from it.

The kidneys (and other organs) show many other examples of self‐organization in their development, including the control of stem cell replacement vs. differentiation, the polarization of epithelia, the organization of blood vessels to serve tissues that secrete chemoattractants such as VEGF, etc. In general, these have been identified by a combination of perturbing the system to show adaptation, identifying ligand‐receptor pairs that might mediate organization, and perturbing their expression pattern to show the expected effect on patterning of the organ. These experimental steps may proceed in a different order, possible signalling molecules being identified first, for example, but the net effect on understanding is the same.

**Figure 3:**
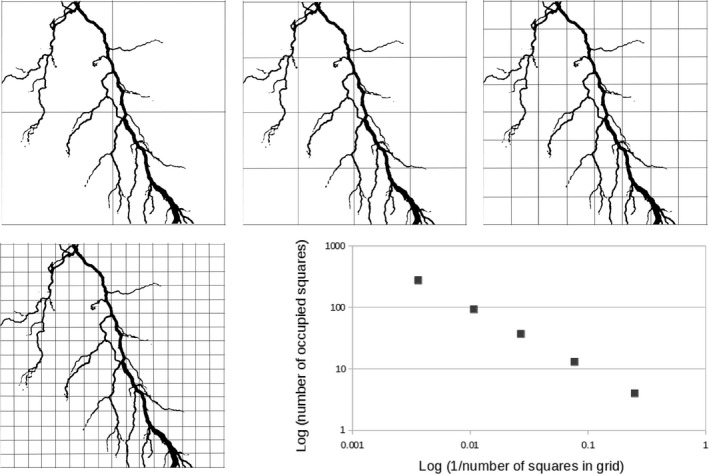
A theoretical approach to seek evidence for self‐organization, by seeking fractal geometry, which is alleged to be associated with self‐organizing systems (but see main text for cautionary notes). The subject of this illustration is a lightning bolt, shown black‐on‐white for clarity, and analysed in two dimensions. The object is viewed under grids of increasing resolution, and in each case the number of squares containing any part of the subject is counted. A graph is then plotted of the log of the number of squares occupied vs. the log of the proportion of the grid occupied by a single square. Fractal structures yield a straight descending line (the gradient of which is related to the fractal dimension): this graph was generated from the image shown. Exactly the same idea can be applied to 3D data (with cubes rather than squares).

In addition to experimental approaches, a theoretical method for detecting self‐organization has been suggested. The topologies of systems that are known to arise by bottom‐up self‐organization rather than top‐down control are characterized by power‐laws. Examples include the Internet, the air transport network, the meanderings of a river and the forking of lightning (reviewed by Turcotte & Rundle, 2002). Many biological systems that are assumed to have arisen by bottom‐up, evolutionary processes rather than centralized control, such as protein networks and metabolic networks, also show power‐law topologies (e.g. a log plot of the number of proteins that interact with a particular protein, vs. the number of proteins with that number of interactions, plots as a descending straight line). Several authors have therefore suggested that self‐organization in development might be identified by seeking power‐law topologies (Kurakin, 2005; Tiraihi et al. 2011). Some features of systems known, through experiment, to self‐organize do show power‐law behaviour: imaging the anatomy of a branching epithelial or vascular tree using voxels of a range of dimensions, assessing the fraction of voxels containing at least some of the tree in each case, and log‐plotting the occupancy vs. voxel size yields a power‐law line, at least until the voxels become smaller than the smallest tubules (see Fig. 3 for an illustration of this method).

There are, however, problems with the approach. The idea that, if something shows a power‐law, top‐down control must be rejected, is unsound. Many structures produced entirely predictably by very precise top‐down mechanisms can also follow power‐laws. An example is Cantor dust: to produce this, draw a line 1 m long, erase the middle third, now erase the middle third from the two lines you have left, and so on *ad infinitum*: a log plot of the length of line vs. the number of lines at that length yields a power‐law. There is nothing self‐organizing about the generation of Cantor dust – it is done by an external agent following precise rules to the letter: this fact alone should caution against assuming that power‐law structures must arise by self‐organization. Also, the association between power‐laws and self‐organization in biological systems often has circular character: we do not actually *know* how protein and metabolic networks with their power‐laws evolved, we just assume that they used only bottom‐up processes and so use this association to bolster the power‐law‐implies‐bottom‐up relationship. Finally, and practically, it is difficult to know what to measure in an embryo to get the data that can be tested for a power‐law, without falling into the trap of data‐dredging until a straight line is found. This approach to identifying self‐organization should therefore be used only with great caution.

## Putting self‐organization to practical use

One of the ways in which basic anatomical and embryological knowledge can be used for practical purposes is in guiding tissue engineering, the attempt to turn progenitor cells into functional tissues. Most current approaches to tissue engineering involve the engineer placing cells where they are needed for the final structure, either by making a hydrogel scaffold and seeding it with cells (Hockaday et al. 2012) or by bio‐printing suspended cells into a defined 3D shape (Nishiyama et al. 2009; Möller et al. 2017). These approaches have proved effective for tissues such as cartilage that involve only a few cell types, that are relatively homogenous and that are matrix‐rich. Tissues such as kidney, which contain dozens of cell types that must be in a very precise arrangement pose more of a problem, especially when it is borne in mind that small faults in the physical relationships of their cells, for example the failure of podocyte foot process interdigitation, can be the reason that someone's kidneys fail in the first place (reviewed by Ranganathan, 2016). At least with current technologies, 3D printing of a kidney is quite impractical. Can self‐organization provide a more effective alternative?

This question has been explored by obtaining renogenic stem cells by disaggregating the presumptive kidney region of E11.5 mouse embryos, re‐aggregating them by centrifugation, and assessing their ability to re‐organize in culture (Unbekandt & Davies, 2010). Provided they were protected from apoptosis by the inclusion of a Rho kinase (ROCK) inhibitor for the first 24 h of culture, the random mix of cells underwent a phase transition: the epithelial (ureteric bud) cells coalesced to form clumps that polarized into cysts then elongated into branching tubules, while the mesenchymal cells near these tubules underwent mesenchymal‐to‐epithelial transition to form nephrons in a manner similar to that seen *in vivo*. This morphogenesis was accompanied by cell differentiation, with markers of each tubular segment being detectable by immunostaining and reverse transcriptase‐polymerase chain reaction. Functional tests of active proximal tubule organic anion and cation transport were positive, with both transporter expression and drug sensitivity data confirming that the normal channels were being used (Lawrence et al. 2015). Furthermore, transplantation of these rudiments into immune‐deficient rat recipients resulted in their being vascularized and forming glomeruli capable of passing traces of fluorescent albumen from the blood into the urinary space, from which it was then recovered by proximal tubule cells (Xinaris et al. 2012).

## Limits to the self‐organization approach: the need for induced symmetry‐breaking

**Figure 4:**
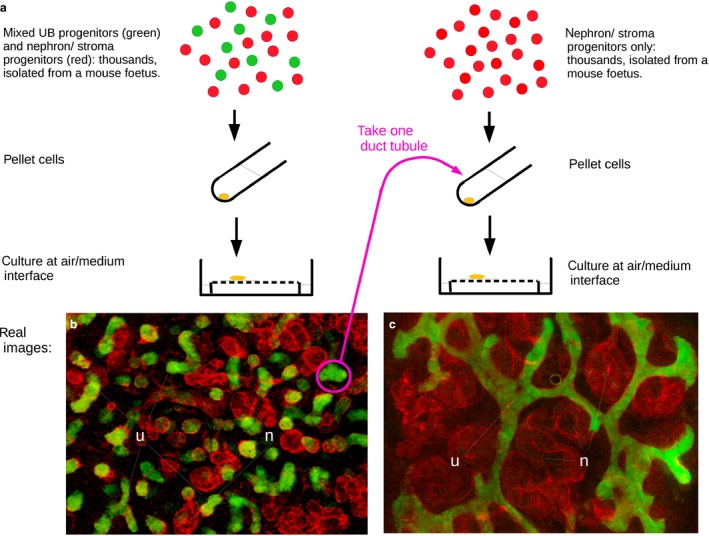
Self‐organizing kidney rudiments, with and without experimenter‐imposed symmetry‐breaking. (a) Depicts the experimental methods of the Unbekandt (left column) and Ganeva (right column) techniques. (b) The result of reaggregating *ex‐fetu* mouse renogenic stem cells; small ureteric bud tubules, some branched, form, scattered throughout the culture (green: stained for the ureteric bud marker calbindin‐D‐28k), and nephrons in their early stages of development (red only: laminin) form among them. (c) The result of taking a single ureteric bud tubule from a culture as in (a), and reaggregating it with *ex‐fetu* renogenic stem cells except those that can make ureteric bud. With the only ureteric bud progenitors in the system in one connected tubule, only a single ureteric bud/collecting duct forms, nephrons forming next to them and connecting to them. This illustrates how simple experimenter‐applied symmetry‐breaking can mimic asymmetrical influences in real embryogenesis and produce more realistic organoids.

The renal organoids described above contain appropriate cell types, and they have normal micro‐scale anatomy in the sense of having tubular, segmented nephrons connected to collecting duct tubules. A high‐magnification image of one might well be mistaken for a foetal kidney, but a low‐power could not, because the larger scale structure of a normal kidney is entirely missing and the organoid is symmetrical (in the sense of being the same everywhere with no special places or directions: Fig. 4a). There is no proper cortex‐medulla division, nephrons are not orientated with respect to any axis, there are no loops of Henle and the collecting duct system appears as a series of scattered tubules and treelets instead of one connected tree ramifying from a ureter ‘trunk’. This latter point is the most critical, because normal renal anatomy is organized primarily by the development of the ureteric bud/collecting duct tree. In an embryo, the ureteric bud enters the kidney‐forming area (metanephrogenic mesenchyme) from the outside as one single tube entering at one place, breaking the symmetry of the system. Can the limited success of self‐organization in the organoid be explained by its being too symmetrical?

This idea was addressed by Ganeva et al. (2011), who designed a serial‐reaggregation experiment. The first aggregation proceeded exactly as in the Unbekandt system described above. The second took one ureteric bud/collecting duct tubule from the first aggregation and combined it with mesenchymal stem cells from the renogenic area of a second embryo, but *not* with any more ureteric bud cells. In this second aggregate, the ureteric bud/collecting duct system grew and a single, coherent tree, with nephrons aligning to it properly (Fig. 4b). In the best culture system developed so far (Chang & Davies, 2012), the rudiments developed distinct medullary and cortical zones, and loops of Henle were seen to extend from the cortex to the medulla and back, as they should.

This engineered kidney was much more realistic than the simple organoid, but the collecting duct tree, while coherent, was symmetrical with no unique ureter to lead urine away. In normal development, the ureteric bud crosses a zone of peri‐Wolffian mesenchyme on its way from its point of emergence from the Wolffian duct to its invasion of the metanephrogenic mesenchyme in which it ramifies to make the collecting duct. The part of the bud that remains in the peri‐Wolffian mesenchyme becomes the ureter, perhaps because of the different signalling environment of this mesenchyme. This raised the possibility that the ureteric bud in the serial‐reaggragates produced a collecting duct tree that lacked a ureter because the *in vivo* asymmetry of the system, with one zone of the ureteric bud in peri‐Wolffian mesenchyme, was missing. To test this idea, we have recently transplanted a piece of peri‐Wolffian mesenchyme next to just one tubule of the growing tree in a serial re‐aggregate, and showed that this tubule ceases to branch and instead expresses the ureter marker, uroplakin (unpublished data presented in the 2016 Anatomical Society December meeting). The symmetry‐breaking conversion of one tubule of the tree into a ureter‐like uroplakin‐positive, unbranched tube can also be driven by local application of a bead soaked in a defined growth factor normally produced by that mesenchyme.

This example illustrates a general approach for improving the performance of organoid production by self‐organizing systems. Self‐organization is powerful but, in the absence of symmetry‐breaking influences that are present in normal development, it is unlikely to produce correct large‐scale anatomy. Judicious, local application of symmetry‐breaking signals (e.g. the bead above) or techniques (e.g. the use of serial reaggregation to force the system to begin with a single epithelium) may be used to add critical missing information to a self‐organizing system and transform its output from being a mere organoid to being a mini‐organ. The general idea should be equally applicable to other types of organoid.

## Lessons for synthetic biology

The systems discussed so far in this article are the products of natural evolution, operating either in their normal developmental context or in artificial organoids. They are beginning to be joined by synthetic biological systems engineered to perform developmental tasks such as patterning, differentiation and morphogenesis using genetic modules that have been designed rather than evolved. Ideas for this were set out by a range of authors (Sia et al. 2007; Teague et al. 2016) a few years ago in a range of journals (including this one; Davies, 2008) and some systems have now become a reality, many in prokaryotes but some in mammalian systems as well (see Davies, 2017 for a recent review).

Commentators such as Bozorgmehr (2014) have pointed out that the most interesting and effective approaches to synthetic development would be those that produce cell behaviours leading to self‐organization and the emergence of coherent large‐scale structures. Some progress has been made in this direction by constructing a patterning system in which randomly mixed cells organize themselves into patches somewhat reminiscent of animal coat markings (Cachat et al. 2016). The system makes use of adhesion‐mediated phase separation. Many decades ago, Steinberg aggregated together cells of different types and showed that they would sort out, apparently with the most adhesive inside and the least adhesive outside (Steinberg, 1963); more recently he used cells engineered to express different levels of the same cadherin to show that this sorting really was predictable on the grounds of adhesion alone (Foty & Steinberg, 2005). Steinberg's systems used small numbers of cells with much freedom to move, and the phase separation could easily run to completion as it does, for example, in a beaker containing oil and water. We reasoned that, in a more constrained system, phase separation would tend to be trapped in local energy minima in which cells separate somewhat to form patches but these patches cannot easily coalesce without cells invading patches of the other cell type, which would increase the contact area between different cell types and be energetically unfavourable. The rich patterns caused by constrained phase separation of oil and water in a very shallow puddle are examples of this effect. Engineering non‐adhesive cells to express either E‐ or P‐cadherin on command, making a random mix of those cells, and then inducing cadherin expression, resulted in the formation of patches or spots (depending on the ratios of the E‐ and P‐cadherin expressing cells; Cachat et al. 2016).

Patterning is only the first stage: what needs to be done now is to couple patterning to the subsequent activation of distinct morphogenetic behaviours in the different patches/lines/spots of cells so that, as in natural development, patterning is the prelude to the creation of physical structure. Possible simple morphogenetic effectors, for first proof‐of‐concept experiments, might be apoptosis of one type of cell (e.g. those in patches, to leave a net) or apical constriction to deform a cell sheet into a corrugated structure.

## Feedback of a different kind: implications for basic research

So far in this article, the limitations of self‐organization have been considered only negatively, as a problem for tissue engineers wanting to use self‐organization to make organoids. Examples were given of how large‐scale features were missing from self‐organized organoids because a critical symmetry‐breaking influence, present in natural embryonic development, was missing and had to be replaced by artificial manipulation. These limitations can be very helpful, however, in the context of basic research, for each failure of realism in a self‐organizing organoid points to the necessity of a feature of natural embryogenesis that would normally provide information. Comparison of development in embryonic organ rudiments and organoids can lead an experimenter to rapid identification of the influences that normally shape organ‐scale anatomy. Finding these will increase our understanding of normal development and, perhaps, identify very specific regulators of organ‐scale anatomy for which small evolutionary change might have a major effect on resulting morphology.

## References

[joa12691-bib-0001] Barak H , Huh SH , Chen S , et al. (2012) FGF9 and FGF20 maintain the stemness of nephron progenitors in mice and man. Dev Cell 22, 1191–1207. 22698282 10.1016/j.devcel.2012.04.018 PMC3376351

[joa12691-bib-0002] Batsivari A , Rybtsov S , Souilhol C , et al. (2017) Understanding hematopoietic stem cell development through functional correlation of their proliferative status with the intra‐aortic cluster architecture. Stem Cell Reports 8(6), 1549–1562. 28479304 10.1016/j.stemcr.2017.04.003 PMC5469869

[joa12691-bib-0003] Bertrand MM , Delmond L , Mazars R , et al. (2014) Is low tie ligation truly reproducible in colorectal cancer surgery? Anatomical study of the inferior mesenteric artery division branches. Surg Radiol Anat 36, 1057–1062. 24633578 10.1007/s00276-014-1281-y

[joa12691-bib-0004] Bozorgmehr JEH (2014) The role of self‐organization in developmental evolution. Theory Biosci 133, 145–163. 24737046 10.1007/s12064-014-0200-4

[joa12691-bib-0005] Braakmana R , Follows MJ , Chisholm SW (2017) Metabolic evolution and the self‐organization of ecosystems. Proc Natl Acad Sci USA E3091–E3100. doi/10.1073/pnas.1619573114 28348231 10.1073/pnas.1619573114 PMC5393222

[joa12691-bib-0006] Cachat E , Liu W , Martin KC , et al. (2016) 2‐ and 3‐dimensional synthetic large‐scale *de novo* patterning by mammalian cells through phase separation. Sci Rep 6, 20 664. 26857385 10.1038/srep20664 PMC4746622

[joa12691-bib-0007] Chang CH , Davies JA (2012) An improved method of renal tissue engineering, by combining renal dissociation and reaggregation with a low‐volume culture technique, results in development of engineered kidneys complete with loops of Henle. Nephron Exp Nephrol 121, e79–e85. 23235540 10.1159/000345514

[joa12691-bib-0008] Chinwalla AT , Cook LL , Delehaunty KD et al. (hundreds of authors) 2002) Initial sequencing and comparative analysis of the mouse genome. Nature 420, 520–562. 12466850 10.1038/nature01262

[joa12691-bib-0009] Cockman ME , Masson N , Mole DR , et al. (2000) Hypoxia inducible factor‐alpha binding and ubiquitylation by the von Hippel‐Lindau tumor suppressor protein. J Biol Chem 275, 25 733–25 741. 10.1074/jbc.M002740200 10823831

[joa12691-bib-0010] Dăescu E , Zăhoi DE , Motoc A , et al. (2012) Morphological variability of the renal artery branching pattern: a brief review and an anatomical study. Rom J Morphol Embryol 53(2), 287–291. 22732797

[joa12691-bib-0011] Davies JA (2005) Mechanisms of morphogenesis. London: Academic Press.

[joa12691-bib-0800] Davies JA (2008) Synthetic morphology: prospects for engineered, self‐constructing anatomies. J Anat. 212(6), 707–719. 18510501 10.1111/j.1469-7580.2008.00896.x PMC2423395

[joa12691-bib-0012] Davies J (2017) Using synthetic biology to explore principles of development. Development 144(7), 1146–1158. 28351865 10.1242/dev.144196

[joa12691-bib-0013] Davies JA , Garrod DR (1995) Induction of early stages of kidney tubule differentiation by lithium ions. Dev Biol 167, 50–60. 7851662 10.1006/dbio.1995.1006

[joa12691-bib-0015] Davies JA , Hohenstein P , Chang CH , et al. (2014) A self‐avoidance mechanism in patterning of the urinary collecting duct tree. BMC Dev Biol 14, 35. 25205115 10.1186/s12861-014-0035-8 PMC4448276

[joa12691-bib-0016] De Palo G , Yi D , Endres RG (2007) A critical‐like collective state leads to long‐range cell communication in Dictyostelium discoideum aggregation. PLoS Biol 15(4), e1002602. |journal.pbio.1002602. 10.1371/journal.pbio.1002602 PMC5396852 28422986

[joa12691-bib-0017] Ezkurdia I , Juan D , Rodriguez JM , et al. (2014) Multiple evidence strands suggest that there may be as few as 19,000 human protein‐coding genes. Hum Mol Genet 23, 5866–5878. 24939910 10.1093/hmg/ddu309 PMC4204768

[joa12691-bib-0018] Forsythe JA , Jiang BH , Iyer NV , et al. (1996) Activation of vascular endothelial growth factor gene transcription by hypoxia‐inducible factor 1. Mol Cell Biol 16, 4604–4613. 8756616 10.1128/mcb.16.9.4604 PMC231459

[joa12691-bib-0019] Foty RA , Steinberg MS (2005) The differential adhesion hypothesis: a direct evaluation. Dev Biol 278, 255–263. 15649477 10.1016/j.ydbio.2004.11.012

[joa12691-bib-0020] Ganeva V , Unbekandt M , Davies JA (2011) An improved kidney dissociation and reaggregation culture system results in nephrons arranged organotypically around a single collecting duct system. Organogenesis 7(2), 83–87. 21386662 10.4161/org.7.2.14881 PMC3142442

[joa12691-bib-0021] Ghosh SK , Biswas S , Sharma S , et al. (2017) An anatomical study of wormian bones from the eastern part of India: is genetic influence a primary determinant of their morphogenesis? Anat Sci Int 92, 373–382. 27038026 10.1007/s12565-016-0342-1

[joa12691-bib-0022] Gordon D , Bernheim‐Groswasser A , Keasar C , et al. (2012) Hierarchical self‐organization of cytoskeletal active networks. Biol Phys 9(2), 026005. (9 pp). 10.1088/1478-3975/9/2/026005 22476003

[joa12691-bib-0023] Green JBA , Dominguez I , Davidson LA (2004) Self‐organization of vertebrate mesoderm based on simple boundary conditions. Dev Dynam 231, 576–581. 10.1002/dvdy.20163 15376320

[joa12691-bib-0024] Henry BM , Vikse J , Graves MJ , et al. (2016) Extralaryngeal branching of the recurrent laryngeal nerve: a meta‐analysis of 28,387 nerves. Langenbecks Arch Surg 401(7), 913–923. 27251487 10.1007/s00423-016-1455-7 PMC5086344

[joa12691-bib-0025] Herrera LM , Strapasson RA , Mazzilli LE , et al. (2017) Differentiation between palatal rugae patterns of twins by means of the Briñón method and an improved technique. Braz Oral Res 31, e9. 28327781 10.1590/1807-3107BOR-2017.vol31.0009

[joa12691-bib-0026] Hess L (1946) Ossicula wormiana. Hum Biol 18, 61–80. 21026244

[joa12691-bib-0027] Hockaday LA , Kang KH , Colangelo NW , et al. (2012) Rapid 3D printing of anatomically accurate and mechanically heterogeneous aortic valve hydrogel scaffolds. Biofabrication 4, 035005. 22914604 10.1088/1758-5082/4/3/035005 PMC3676672

[joa12691-bib-0028] Hölldobler B , Wilson EO (2008) The Superorganism: The Beauty, Elegance, and Strangeness of Insect Societies. New York: W W Norton.

[joa12691-bib-0029] International Human Genome Sequencing Consortium (2001) Initial sequencing and analysis of the human genome. Nature 409, 860–921. 11237011 10.1038/35057062

[joa12691-bib-0030] Jainchill J , Saxen L , Vainio T (1964) Studies on kidney tubulogenesis I: the effect of actinomycin D on tubulogenesis *in vitro* . J Embryol Exp Morphol 12, 597–607. 14251472

[joa12691-bib-0031] Karsenti E (2008) Self‐organization in cell biology: a brief history. Nat Rev Mol Cell Biol 9, 255–262. 18292780 10.1038/nrm2357

[joa12691-bib-0032] Kates WR , Ikuta I , Burnette CP (2009) Gyrification patterns in monozygotic twin pairs varying in discordance for autism. Autism Res 2(5), 267–278. 19890876 10.1002/aur.98

[joa12691-bib-0033] Kücken M , Champod C (2013) Merkel cells and the individuality of friction ridge skin. J Theor Biol 21(317), 229–237. 10.1016/j.jtbi.2012.10.009 23079286

[joa12691-bib-0034] Kurakin A (2005) Self‐organization versus Watchmaker: stochastic dynamics of cellular organization. Biol Chem 386, 247–254. 15843170 10.1515/BC.2005.030

[joa12691-bib-0035] Kuure S , Popsueva A , Jakobson M , et al. (2007) Glycogen synthase kinase‐3 inactivation and stabilization of beta‐catenin induce nephron differentiation in isolated mouse and rat kidney mesenchymes. J Am Soc Nephrol 218, 1130–1139. 10.1681/ASN.2006111206 17329570

[joa12691-bib-0036] Lavington E , Cogni R , Kuczynski C , et al. (2014) A small system–high‐resolution study of metabolic adaptation in the central metabolic pathway to temperate climates in Drosophila melanogaster. Mol Biol Evol 31, 2032–2041. 24770333 10.1093/molbev/msu146 PMC4104311

[joa12691-bib-0037] Lawrence ML , Chang CH , Davies JA (2015) Transport of organic anions and cations in murine embryonic kidney development and in serially‐reaggregated engineered kidneys. Sci Rep 13(5), 9092. 10.1038/srep09092 PMC4357899 25766625

[joa12691-bib-0038] Liu Y , Cox SR , Morita T , et al. (1995) Hypoxia regulates vascular endothelial growth factor gene expression in endothelial cells. Identification of a 5’ enhancer. Circ Res 77, 638–643. 7641334 10.1161/01.res.77.3.638

[joa12691-bib-0039] Marco‐Clement I , Martinez‐Barco A , Ahumada N , et al. (2016) Anatomical variations of the celiac trunk: cadaveric and radiological study. Surg Radiol Anat 38, 501–510. 26267305 10.1007/s00276-015-1542-4

[joa12691-bib-0040] Martin KC , Yuan X , Stimac G , et al. (2017) Symmetry‐breaking in branching epithelia: cells on micro‐patterns under flow challenge the hypothesis of positive feedback by a secreted autocrine inhibitor of motility. J Anat. 230(6), 766–774. 28369863 10.1111/joa.12599 PMC5442143

[joa12691-bib-0041] Matejcík V (2010) Anatomical variations of lumbosacral plexus. Surg Radiol Anat 32(4), 409–414. 19696958 10.1007/s00276-009-0546-3

[joa12691-bib-0042] Maxwell PH , Wiesener MS , Chang GW , et al. (1999) The tumour suppressor protein VHL targets hypoxia‐inducible factors for oxygen‐dependent proteolysis. Nature 399, 271–275. 10353251 10.1038/20459

[joa12691-bib-0043] Meinhardt H , Gierer A (2000) Pattern formation by local self‐activation and lateral inhibition. BioEssays 22, 753–760. 10918306 10.1002/1521-1878(200008)22:8<753::AID-BIES9>3.0.CO;2-Z

[joa12691-bib-0044] Millauer B , Wizigmann‐Voos S , Schnurch H , et al. (1993) High affinity VEGF binding and developmental expression suggest Flk‐1 as a major regulator of vasculogenesis and angiogenesis. Cell 72, 835–846. 7681362 10.1016/0092-8674(93)90573-9

[joa12691-bib-0045] Misteli T (2001) The concept of self‐organization in cellular architecture. J Cell Biol 155, 181–185. 11604416 10.1083/jcb.200108110 PMC2198832

[joa12691-bib-0046] Möller T , Amoroso M , Hägg D , et al. (2017) *In vivo* chondrogenesis in 3D bioprinted human cell‐laden hydrogel constructs. Plast Reconstr Surg Glob Open 5(2), e1227. 28280669 10.1097/GOX.0000000000001227 PMC5340484

[joa12691-bib-0047] Montesano R , Schaller G , Orci L (1991) Induction of epithelial tubular morphogenesis *in vitro* by fibroblast‐derived soluble factors. Cell 66, 697–711. 1878968 10.1016/0092-8674(91)90115-f

[joa12691-bib-0048] Munro DAD , Hohenstein P , Davies JA (2017) Cycles of vascular plexus formation within the nephrogenic zone of the developing mouse kidney. Sci Rep. 7(1), 3273. 28607473 10.1038/s41598-017-03808-4 PMC5468301

[joa12691-bib-0049] Muthukrishnan SD , Yang X , Friesel R , et al. (2015) Concurrent BMP7 and FGF9 signalling governs AP‐1 function to promote self‐renewal of nephron progenitor cells. Nat Commun 6, 10 027. 10.1038/ncomms10027 PMC4686668 26634297

[joa12691-bib-0050] Nelson CM , Vanduijn MM , Inman JL , et al. (2006) Tissue geometry determines sites of mammary branching morphogenesis in organotypic cultures. Science 314, 298–300. 17038622 10.1126/science.1131000 PMC2933179

[joa12691-bib-0051] Nishiyama Y , Nakamura M , Henmi C , et al. (2009) Development of a three‐dimensional bioprinter: construction of cell supporting structures using hydrogel and state‐of‐the‐art inkjet technology. J Biomech Eng 131, 035001. 19154078 10.1115/1.3002759

[joa12691-bib-0052] Ranganathan S (2016) Pathology of podocytopathies causing nephrotic syndrome in children. Front Pediatr 24, 32. 10.3389/fped.2016.00032 PMC4814732 27066465

[joa12691-bib-0053] Reinhoff WF (1922) Development and growth of the metanephros or permanent kidney in chick embryos. Johns Hopkins Hospital Bulletin 33, 392–406.

[joa12691-bib-0054] Röder PV , Wu B , Liu Y , et al. (2016) Pancreatic regulation of glucose homeostasis. Exp Mol Med 48(3), e219. 26964835 10.1038/emm.2016.6 PMC4892884

[joa12691-bib-0055] Rodriguez‐Brenes IA , Wodarz D , Komarova NL (2013) Minimizing the risk of cancer: tissue architecture and cellular replication limits. J R Soc Interface 10, 20130410. 23825115 10.1098/rsif.2013.0410 PMC3730689

[joa12691-bib-0056] Romanovsky AA (2007) Thermoregulation: some concepts have changed. Functional architecture of the thermoregulatory system. Am J Physiol Regul Integr Comp Physiol 292, R37–R46. 17008453 10.1152/ajpregu.00668.2006

[joa12691-bib-0057] Schilling CH , Schuster S , Palsson BO , et al. (1999) Metabolic pathway analysis: basic concepts and scientific applications in the post‐genomic era. Biotechnol Prog 15, 296–303. 10356246 10.1021/bp990048k

[joa12691-bib-0058] Schreiner KE (1902) Uber die Entwicklung der Amniotenniere. Zeitsch f wiss Zool 71, 1–188.

[joa12691-bib-0059] Sia SK , Gillette BM , Yang GJ (2007) Synthetic tissue biology: tissue engineering meets synthetic biology. Birth Defects Res C Embryo Today 81, 354–361. 18228264 10.1002/bdrc.20105

[joa12691-bib-0060] Silva PS , Vilarinho A , Carvalho B , et al. (2014) Anatomical variations of the vein of Labbé: an angiographic study. Surg Radiol Anat 36(8), 769–773. 24531417 10.1007/s00276-014-1264-z

[joa12691-bib-0061] Srihari SN , Srinivasan H , Fang G (2008) Discriminability of fingerprints of twins. J Forensic Identification 58, 109–127.

[joa12691-bib-0801] Steinberg MS (1963) Reconstruction of tissues by dissociated cells. Some morphogenetic tissue movements and the sorting out of embryonic cells may have a common explanation. Science 141(3579), 401–408. 13983728 10.1126/science.141.3579.401

[joa12691-bib-0062] Sweeney D , Lindström N , Davies JA (2008) Developmental plasticity and regenerative capacity in the renal ureteric bud/collecting duct system. Development 2135, 2505–2510. 10.1242/dev.022145 18579677

[joa12691-bib-0063] Tai G , Hohenstein P , Davies JA (2013) FAK‐Src signalling is important to renal collecting duct morphogenesis: discovery using a hierarchical screening technique. Biol Open 2, 416–423. 23616926 10.1242/bio.20133780 PMC3625870

[joa12691-bib-0064] Takeichi M (2011) Self‐organization of animal tissues: Cadherin‐mediated processes. Dev Cell 21, 24–26. 21763603 10.1016/j.devcel.2011.06.002

[joa12691-bib-0065] Teague BP , Guye P , Weiss R (2016) Synthetic Morphogenesis. Cold Spring Harb Perspect Biol 8(9), a023929. 27270296 10.1101/cshperspect.a023929 PMC5008072

[joa12691-bib-0066] Tiraihi A , Tiraihi M , Tiraihi T (2011) Self‐organization of developing embryo using scale‐invariant approach. Theor Biol Med Model 8, 24. art 17. 21635789 10.1186/1742-4682-8-17 PMC3126770

[joa12691-bib-0067] Tomaszewski KA , Henry BM , Vikse J , et al. (2017) Variations in the origin of the deep femoral artery: a meta‐analysis. Clin Anat 30, 106–113. 26780216 10.1002/ca.22691

[joa12691-bib-0068] Townsend GC , Richards L , Hughes T , et al. (2005) Epigenetic influences may explain dental differences in monozygotic twin pairs. Aust Dent J 50(2), 95–100. 16050088 10.1111/j.1834-7819.2005.tb00347.x

[joa12691-bib-0069] Trowell OA (1954) A modified technique for organ culture *in vitro* . Exp Cell Res, 6, 246–248. 13142005 10.1016/0014-4827(54)90169-x

[joa12691-bib-0070] Turcotte DL , Rundle JB (2002) Self‐organized complexity in the physical, biological, and social sciences. Proc Natl Acad Sci USA 99(Suppl 1), 2463–2465. 11875195 10.1073/pnas.012579399 PMC128561

[joa12691-bib-0071] Turing AM (1953) The chemical basis of morphogenesis. Philos Trans R Soc Lond B Biol Sci 237, 37–72.

[joa12691-bib-0072] Unbekandt M , Davies JA (2010) Dissociation of embryonic kidneys followed by reaggregation allows the formation of renal tissues. Kidney Int 77, 407–416. 20016472 10.1038/ki.2009.482

[joa12691-bib-0073] Wolf KT , Brokaw EJ , Bell A , et al. (2016) Variant inferior alveolar nerves and implications for local anesthesia. Anesth Prog 63, 84–90. 27269666 10.2344/0003-3006-63.2.84 PMC4896047

[joa12691-bib-0074] Xinaris C , Benedetti V , Rizzo P , et al. (2012) *In vivo* maturation of functional renal organoids formed from embryonic cell suspensions. J Am Soc Nephrol 23, 1857–1868. 23085631 10.1681/ASN.2012050505 PMC3482737

[joa12691-bib-0075] Yu Y , Sato JD (1999) MAP kinases, phosphatidylinositol 3‐kinase, and p70 S6 kinase mediate the mitogenic response of human endothelial cells to vascular endothelial growth factor. J Cell Physiol 178, 235–246. 10048588 10.1002/(SICI)1097-4652(199902)178:2<235::AID-JCP13>3.0.CO;2-S

